# Hypertension Knowledge, Attitude, and Practice in Adult Hypertensive Patients at a Tertiary Care Hospital in Sri Lanka

**DOI:** 10.1155/2020/4642704

**Published:** 2020-10-21

**Authors:** Udaya Ralapanawa, Kameera Bopeththa, Noorika Wickramasurendra, Sampath Tennakoon

**Affiliations:** ^1^Department of Medicine, University of Peradeniya, Peradeniya, Sri Lanka; ^2^Emergency Medicine, Teaching Hospital, Peradeniya, Sri Lanka; ^3^Community Medicine, Faculty of Medicine, University of Peradeniya, Peradeniya, Sri Lanka

## Abstract

**Objective:**

Hypertension (HTN) remains a major risk factor for cardiovascular diseases globally. Despite considerable improvement in increasing awareness, treatment, and control of HTN, undiagnosed and uncontrolled HTN remains a major public health challenge. Our focus was on studying the knowledge, attitude, and practice regarding HTN in adult hypertensive patients at a tertiary care hospital in Sri Lanka. *Methodology*. A descriptive study on knowledge, attitude, and practice of hypertensive patients on antihypertensive medications attending the hypertensive clinic was carried out from January 2016 to June 2016 at THP.

**Results:**

The study was on a total of 371 hypertensive patients comprising 253 (68.2%) females and 118 (31.8%) males. Among the total participants, 12 (3.2%), all females, had never been to school. About half of them (47.7%) had not even reached GCE (ordinary level). More than two-thirds (77%) of the study population were aware of the complications of HTN as they were informed by a health care team member. About 74% of them were taking all their prescribed medications. Almost all (95%) patients had checked their blood pressure (BP) during the previous 12 months, and almost the same percentage had visited their doctor for BP checkups every 1–3 months.

**Conclusion:**

Our patients were well aware of the importance of regular follow-ups and also knowledgeable about the complications related to HTN. Almost 75% of the patients had optimum drug compliance. It was revealed that forgetfulness was the frequent cause for poor drug compliance.

## 1. Introduction

HTN remains a major risk factor for cardiovascular diseases around the world [1–4]. Globally, cardiovascular disease accounts for approximately 17 million deaths a year, nearly one-third of the total. Of these, complications of HTN account for 9.4 million deaths worldwide annually [5]. Despite considerable improvement in increasing the awareness, treatment, and control of HTN, undiagnosed and uncontrolled HTN remains a public health challenge [6–8]. It disproportionately affects populations in low- and middle-income countries, with consequences such as stroke, kidney failure, peripheral vascular disease, and premature disability [9–11]. Similarly, HTN is frequently seen among hospital patients in the Republic of Sri Lanka. However, the prevalence of HTN in Sri Lanka is not known [12, 13].

HTN presents a major area of intervention because it is a frequently occurring condition that is amenable to control through both nonpharmacological lifestyle factors and pharmacological treatment [14]. While antihypertensive medications have been used for blood pressure control, there has been increasing emphasis on the prevention and treatment of HTN by nonpharmacological means termed lifestyle modifications [15]. The recommended lifestyle measures that have been shown to be capable of reducing blood pressure include (i) salt restriction, (ii) moderation of alcohol consumption, (iii) high consumption of vegetables and fruits and low-fat and other types of diet, (iv) weight reduction and maintenance, and (v) regular physical exercise. Hypertensive patients irrespective of their stage or grade should be motivated to adopt these measures. Motivating patients to implement lifestyle changes is probably one of the most difficult aspects of managing HTN [16]. A KAP survey means knowledge, attitude, and practice. KAP questions tend to reveal not only characteristic traits in knowledge, attitude, and behaviors about health but also the idea that each person has of the disease. These factors are often the source of misunderstandings. The obstacle to change may be lack of knowledge [17]. Recent reports have suggested that HTN knowledge is related to blood pressure control [18]. The importance of HTN awareness and knowledge and the potential impact of BP education programs have been reported on recently [19–21]. Although the outcome of a KAP study seems simple, the results of the study can have a huge impact on the local community. As the KAP study explores what is known and what is done in relation to a health care-related objective which is about hypertension in this study, the results will reveal the baseline information of the community and may reveal the misconceptions or misbehaviors in relation to practice of hypertension. It is very important to identify these facts as these directly influence the future health care-related interventions. Moreover, results of the KAP study establish reference values on various health care parameters for use in future assessments. Also, aspects assessed by the KAP study are not uniform to the entire communities. Therefore, results of the KAP study derived based on Western data cannot be applied to our local community. In summary, it is justifiable to conduct a study on assessment of knowledge, attitude, and practice in relation to hypertension in a local community as this will reveal important unknown data on hypertension to guide future research studies and health-related interventions.

This study was conducted to study the knowledge, attitude, and practice of HTN in adult hypertensive patients at Teaching Hospital Peradeniya (THP). This is aimed at the potential new development of interventional strategies to reduce HTN-related morbidity and mortality.

## 2. Materials and Methods

### 2.1. Study Design and Site

A descriptive study on knowledge, attitude, and practice of hypertensive patients on antihypertensive medications attending the hypertensive clinic was carried out from January 2016 to June 2016 at THP. This is one of the largest tertiary care hospitals in the central province of Sri Lanka. The approval for this study was obtained from the Ethical Review Committee of the Faculty of Medicine, Peradeniya, and the administration of the hospital.

### 2.2. Inclusion and Exclusion Criteria

Patients who were on antihypertensive medications for more than six months with uninterrupted clinic attendance, with or without comorbidities of both sexes, were included in the study. Excluded from the study were pregnant patients.

### 2.3. Sample Size and Sampling

Following application of inclusion and exclusion criteria, a total of 371 patients representing all patients attending the clinic during the study period were enrolled in the study.

### 2.4. Data Collection

The study was conducted within the hypertensive clinic premises. A well-structured, face-to-face, interviewer-administered questionnaire was administered in the local language by four well-trained health care professionals who had previous experience in conducting such studies. Comprehensive sociodemographic information of the participants was gathered at the beginning of the study. There were 22 questions assessing knowledge, attitude, and practice of hypertensive patients. The questionnaire was prepared in the English language and translated into the local language and finalized after pretesting.

### 2.5. Variables and the Method of Verification

Knowledge and attitude questions were posed. Three fixed answering options, “yes,” “no” followed by “uncertain,” were offered. Other close-ended questions especially related to questions assessing practice were organized to offer three to seven response options. If a participant had ever smoked, then they were categorized as smokers, and options were “yes” or “never.” Similar options were offered to those who had imbibed alcohol as well.

### 2.6. Statistical Analysis

Data were entered in an electronic format and analyzed using SPSS 16 package. A monovariate analysis was performed to describe the study sample, and chi-square and difference between means were used to explore associations between variables. A *p* value less than 0.001 was considered statistically significant.

## 3. Results

Baseline sociodemographic characteristics of the patients participated in the study are depicted in Table 1. There were a total of 371 hypertensive patients, and among them, 253 (68.2%) were females and 118 (31.8%) were males. The median age of male participants was 65 years and that of female participants was 64 years. The total study population was composed of 91.9% Sinhalese, 2.2% Tamils, 5.7% Muslims, and 0.5% of other ethnic groups. Of the participants, there were 91.9% Buddhists, 0.8% Hindus, 5.7% Muslims, 1.3% Christians, and 0.3% other religions. Among the total participants, 12 (3.2%) had never gone to school, and all were females. About half of them (47.7%) had not studied up to the GCE (ordinary level). Of these, 177 patients, a majority (72.9%) were females. Only 14 (3.8%) patients had stepped beyond the advanced level and showed equal gender distribution. There was a statistically significant association between gender and level of education (*p*=0.013). Of the total participants, 171 (46.1%) were unemployed, and of them, 99.4% were females. There were 83 (22.4%) unskilled workers, 47 (12.7%) skilled workers, 25 (6.7%) business workers, and 45 (12.1%) professionals. Of the professionals, 53.3% were females. There was a statistically significant association between gender and category of occupation (*p* ≤ 0.001). Approximately 50% of patients had a sufficient household monthly income of more than 12,000 rupees. Only 3.5% of participants had incomes less than 3000 rupees. 75% of the patients had never smoked, and 90% of the patients were females. Similarly, 278 (75%) had never ingested alcohol, and out of them, 90% were females. There was a statistically significant association of gender with smoking and alcohol consumption (*p* ≤ 0.001).

According to Table 2, more than half of our study population (60%) had chronic HTN (more than 5 years). Fifty percent reported that they were first diagnosed as having HTN in a tertiary care hospital, and only 6.5% were diagnosed in a primary care centre. Almost all patients (94%) were taking antihypertensive medications to control their HTN. More than two-thirds (77%) of the study population knew of the complications of HTN and were informed by a health care team member. A significant percentage (77%) of patients expressed that strokes were related to HTN. Regarding complications related to HTN, 35% had cardiovascular complications, 5% percent had suffered from strokes, and 3% percent had renal disease. When questioned regarding their family history of HTN, 58% of patients recalled positive family histories. Inquiries regarding lifestyle changes advised by a doctor to lower BP and blood cholesterol level revealed that a considerable percentage (65%) had received such advice. Questions about blood cholesterol level showed that 55% of patients were on medications to lower blood cholesterol levels, and the same percentage of patients had checked their cholesterol levels during the previous 12 months. Inquiries about drug compliance revealed that 74% were taking all prescribed medications, but detailed questioning did not reveal specific reason(s). We asked patients about the follow-up on their HTN. Almost all (95%) patients had checked their BP within the last 12 months, and almost the same percentage of patients had seen their doctor for BP checkup every 1–3 months. Fifty-eight patients had attended our health care institution, and 25% had attended a nearby primary or secondary health care facility for routine follow-up to check their BP.

## 4. Discussion

We conducted a descriptive study to assess the current status of knowledge, attitude, and practice about HTN in adult hypertensive patients at THP. Although globally men have a slightly higher prevalence of HTN than women, women had a higher prevalence of HTN in our study [22]. The literacy rate is also higher in men globally, and our study also showed the same results [23]. Our studies have found that, of 50–60% of the study population, the place of the first diagnosis of HTN and place of regular follow-up was a tertiary care hospital. Uncomplicated hypertensive patients can be easily managed at primary or secondary care hospitals. Reasons for bypassing these levels may be due to developing demand for specialized care and or unavailability of proper referral systems [24]. Therefore, attention must be paid when implementing measures to improve health infrastructure in the future. Our patients were well aware of the importance of regular follow-up and knowledgeable about the complications related to HTN because 95% of patients had checked their BP within the last 12 months with regular 1–3 monthly interval checkups, and over 75% of the patients were aware of the complications related to HTN. We asked patients about advice received from a doctor to change their way of life to lower BP. Almost 70% of patients reported that a doctor had indeed explained the matter to them. To achieve the maximum coverage of patients, we may have to use other sources to disseminate HTN information including mass media as it had been identified as a major source of information [25]. Fifty-three percent of the study population had blood relatives with HTN. This may signify the genetic predisposition of HTN. For early recognition of HTN, we may need to have national level awareness enhancing programs to recognize the at-risk group. To ascertain their attitude towards adherence to taking all prescribed medications, we posed questions about their drug compliance. Almost 75% of patients had optimum drug compliance. Further exploration for reasons of poor drug compliance revealed forgetfulness to be the most frequent cause. Forgetfulness may not imply patient's poor attitude towards the disease but could be due to various underlying reasons such as lack of knowledge about HTN, lack of adequate guidance, attitudes regarding treatment of an often asymptomatic condition, and personal health beliefs [26].

## 5. Conclusions

HTN is a major disease condition leading to multiple comorbidities and eventually death. Preventive medicine plays a significant role in HTN management. Assessment of KAP towards HTN is essentially required for new development of knowledge-, attitude-, and practice-enhancing programs to reduce the burden of HTN in a country. Our study showed overall satisfactory knowledge about complications of HTN and the importance of adherence to close follow-up. Almost two-thirds of the patients had received advice regarding lifestyle changes resulting in a change of practice. Twenty-five percent of the patients had poor drug compliance, and patients' attitudes towards drug compliance need to be further evaluated by future studies.

## 6. Limitations

The current study was restricted to clinic patients and conducted in a single centre. Considering the fact that factors relating to hypertension looked at in this study may vary demographically in other regions of Sri Lanka, making strong generalizations of the findings, sans multicentre study or a meta-analysis of similar data obtained from two or more regions may have limited accuracy.

## Figures and Tables

**Table 1:** Baseline sociodemographic characteristics of the patients participated in the study.

Baseline sociodemographic	Characteristics (*N* = 371)	
Characteristics	Category	*N* (%)
Sex	Female	253 (68.2)
	Male	118 (31.8)

Ethnicity	Sinhala	340 (91.6)
	Tamil	8 (2.2)
	Moor	21 (5.7)
	Others	2 (0.5)

Religion	Buddhist	341 (91.9)
	Hindu	3 (0.8)
	Islam	21 (5.7)
	Christian	5 (1.3)
	Others	1 (0.3)

Level of education	No school	12 (3.2)
	<ordinary level	177 (47.7)
	Ordinary level	103 (27.8)
	O/L-A/L	4 (1.1)
	Advanced level	61 (16.4)
	Beyond A/L	14 (3.8)

Occupation	House work	171 (46.1)
	Unskilled	83 (22.4)
	Skilled	47 (12.70)
	Business	25 (6.7)
	Professional	45 (12.1)

Monthly income (in rupees)	<3000	13 (3.5)
	3000–6000	19 (5.1)
	6000–12000	49 (13.2)
	>12000	185 (49.9)
	Not answered	104 (28)

Ever smoked	Yes	91 (25)
	Never	280 (75)

Ever took alcohol	Yes	93 (25)
	Never	278 (75)

**Table 2:** KAP related to hypertension.

	*N* (%)
How did you come to know about your hypertension?	
In a routine medical clinic	66 (17.8)
Screening programme	29 (7.8)
Emergency service	70 (19)
Others	192 (52)
I do not know	1 (0.3)

When were you diagnosed?	
First time	4 (1.4)
Less than 5 years	129 (34.8)
More than 5 years	224 (60.4)

Where were you first diagnosed as having hypertension?	
Primary health care	24 (6.5)
Physician	59 (16)
Secondary care hospital	84 (22)
Tertiary care hospital	187 (50)
At a pharmacy	
Others	
I do not know	

Where do you go for routine follow-up to check blood pressure?	
Diagnosis on this visit	13 (3.5)
This health care	216 (58)
Nearby primary health care clinic	32 (8.6)
Nearby hospital	54 (15)
Tertiary hospital	22 (6)
I do not do any follow-up	21 (6)

When was your blood pressure last measured by a health care professional?	
Within the past 12 months	354 (95)
1–5 years ago	3 (0.8)
Not within the past 5 years	

How often do you see your doctor for blood pressure checkup?	
Monthly	154 (40.7)
Every 3, 4 months	186 (50)
Every 6 months	4 (1.1)
Once a year	2 (0.5)

When was your blood cholesterol last measured?	
Within the past 12 months	203 (54)
1–5 years ago	83 (22)
Not within the past 5 years	65 (12.5)

Are you taking medications to lower your blood cholesterol level?	
Yes	204 (55)
No	50 (16)
Uncertain	92 (26)

Has a doctor in the past year ordered you to change your way of life to lower blood cholesterol level?	
Yes	242 (65)
No	25 (7)
Uncertain	92 (25)

Are you currently taking aspirin or equivalent to prevent or treat heart disease or stroke?	
yes	139 (38)
No	87 (24)
Uncertain	133 (56)

Are you currently using hormone replacement therapy?	
Yes	4 (1.1)
No	101 (27)
Uncertain	254 (67)
Do you have blood relatives of hypertension?	
Yes	214 (55)
No	33 (9)
Uncertain	92 (24.8)

Have you had any complication from your hypertension?	
No	42 (11.7)
Renal disease	10 (3)
Stroke	19 (5)
Retinopathy	10 (3)
Cardiovascular	131 (35)
Others	1 (0.3)
I do not know	114 (30)

Has a doctor in the past year ordered you to change your way of life to lower your blood pressure?	
Yes	259 (70)
No	18 (5)
Uncertain	83 (22)

Have you been prescribed any medication to lower your blood pressure?	
Yes	348 (94)
No	1 (0.3)
I do not know	10 (3)

Do you take all your prescribed medications?	
Yes	272 (74)
No	1 (0.3)

If you don't take medications regularly, why don't you take them as directed?	
I cannot afford the cost	2 (0.5)
My medications are not easily available	3 (0.8)
I do not like to take medications	4 (1.1)
I only take them when I feel that I need them	5 (1.3)
I do not like the side effects of the medication	10 (3)
I prefer alternative medicine	1 (0.3)
I forget	46 (12.4)
I do not know	1 (0.3)
Not answered	293 (79)

Are you aware of any complication of hypertension?	
Yes	286 (77)
No	7 (1.9)

If you are aware, have you been informed by a health care professional?	
Yes	278 (75)
No	7 (2)

Have you been told that stroke is related to hypertension?	
Yes	285 (77)
No	7 (2)

## Data Availability

The data of the study are available with the authors and are secured with password protection for which only the authors have access to.

## Abbreviations

BP: Blood pressure
HTN: Hypertension
KAP: Knowledge, attitude, and practice.
